# An artifacts removal post-processing for epiphyseal region-of-interest (EROI) localization in automated bone age assessment (BAA)

**DOI:** 10.1186/1475-925X-10-87

**Published:** 2011-09-28

**Authors:** Hum Yan Chai, Lai Khin Wee, Tan Tian Swee, Sh-Hussain Salleh, Lim Yee Chea

**Affiliations:** 1 Centre for Biomedical Engineering, Faculty of Health Science and Biomedical Engineering, Universiti Teknologi Malaysia, Skudai, 81310, Johor, Malaysia; 2 Biomedical Engineering Group, Institute of Biomedical Engineering and Informatics, Faculty of Computer Science and Automation, Technische Univeristat Ilmenau, Germany

## Abstract

**Background:**

Segmentation is the most crucial part in the computer-aided bone age assessment. A well-known type of segmentation performed in the system is adaptive segmentation. While providing better result than global thresholding method, the adaptive segmentation produces a lot of unwanted noise that could affect the latter process of epiphysis extraction.

**Methods:**

A proposed method with anisotropic diffusion as pre-processing and a novel Bounded Area Elimination (BAE) post-processing algorithm to improve the algorithm of ossification site localization technique are designed with the intent of improving the adaptive segmentation result and the region-of interest (ROI) localization accuracy.

**Results:**

The results are then evaluated by quantitative analysis and qualitative analysis using texture feature evaluation. The result indicates that the image homogeneity after anisotropic diffusion has improved averagely on each age group for 17.59%. Results of experiments showed that the smoothness has been improved averagely 35% after BAE algorithm and the improvement of ROI localization has improved for averagely 8.19%. The MSSIM has improved averagely 10.49% after performing the BAE algorithm on the adaptive segmented hand radiograph.

**Conclusions:**

The result indicated that hand radiographs which have undergone anisotropic diffusion have greatly reduced the noise in the segmented image and the result as well indicated that the BAE algorithm proposed is capable of removing the artifacts generated in adaptive segmentation.

## Introduction

Bone age assessment (BAA) or bone maturity assessment is a clinical application used to evaluate the skeletal development especially in children and adolescents. Due to the inefficiency to describe maturation age using chronological age, the skeletal maturity or skeletal age is utilized as indicator for growth disorders as well as the predictor for final body height [1]. The radiograph of left hand is proven [2] to be a reliable indicator of skeletal maturation and therefore is used as the skeletal to represent the biological maturity depending on features like development of ossification area and calcium position in the ossification area. Diseases of children like endocrine disorders, chromosomal disorders, early sexual maturation, and others [3] can be detected via the discrepancy between the skeletal age and biological age.

Basically there are two major type of evaluation system are being used [4]: the Greulich-Pyle [5] and Tanner-Whitehouse atlas (TW2) [6]. For the Greulich-Pyle method, the physicians compare the patient's hand bone radiograph with the atlas and make the conclusion whereas the TW2 method is a point collection index system. The reliability and efficiency of both methods are frequently debated [7] as they are carried out using visual inspection, highly dependent on the physician knowledge background and perspective and time-consuming [8,9]. Therefore, in recent years, numerous automated system of BAA have been developed especially for TW2 method which is more appropriate for computing purpose [10]. However, the automated system is still under the experimental stage [11] due to the insufficient stability of the system.

Almost all the automated BAA system undergo a pre-processing stage of segmentation with the intent of removing the background, noise, soft-tissue region which contains no pertinence of information that will affect the computerized performance. However most of the conventional methods used are obsolete and unreliable. Besides, most of the researches perform the segmentation after obtaining the region of interest (ROI) to reduce the difficulty of segmentation. In fact, this accuracy and performance of ROI searching can be improved by performing the algorithm after segmenting the hand bone from the soft-tissue region. Being one of the significant initial stages of the system, the output accuracy and effectiveness of segmentation is prominent since the quality of the system output relies heavily on this stage.

The study conducted will focus on the separation of background and soft-tissue region from the hand's skeletal bone: Phalanges, distal phalange, middle phalange, proximal phalange, metacarpus, carpus, hamate, capitates, trapezoid, trapezium, triquetral, lunate, scaphoid, sesamoid bone. The data implemented in the computing analysis are collected from the clinic of University Teknologi Malaysia and also from the Greulich-Pyle atlas.

The main parts of the hand radiograph are the hand bone, soft-tissue region and the background. Therefore, an intuitive approach to segment the bone from the background and soft-tissue region is clustering [12,13]. The classical k-mean clustering, with k equals to two or three, has been adopted to perform the hand bone segmentation in previous literature [13]. However, it is the nature of clustering method in image processing that they do not consider the spatial information of the anatomical pixels. In other words, the segmentation based on classical k-mean is inherently a thresholding method and the only difference between k-mean clustering and thresholding segmentation would be the automated threshold setting property (the unsupervised k-mean possesses the ability to search for a threshold rather than pre-setting it in advance). Nonetheless, the dilemma remains unsolved: the same pixels intensity value in the finger spongy bone (cancellous bone) and the soft-tissue region. It means there is no single threshold value that could completely separate the bone and soft-tissue region in a simultaneous manner. Therefore, it turns out that only two possibilities could occur in the output image: the threshold (k-mean output) is set higher, the cancellous bone and the soft-tissue region are both disappeared in the output image; the threshold is set lower, the cancellous bone and the soft-tissue region are both remained in the output image. Unfortunately, both cases are not desired.

This kind of problem is not unusual. The two possibilities mentioned will impose two impacts on the output image. First, areas disappear and only one of them need to be recovered (cancellous bone); Second, both areas remains and only one of them need to be discarded (soft-tissue region). Previous literatures implemented region growing in solving the problem. Nevertheless, this kind of technique will blur the anatomical edge which will affect the measurement of the anatomical structure in the subsequent parts of the (Computer-aided Diagnosis) CAD system. Therefore, our aim in this paper is to design an automated edge preserving post-processing technique that could simultaneously perform the cancellous bone area recovery and soft-tissue region discard. The performance of this task is further improved by applying the anisotropic diffusion [14] before the clustering segmentation with the intent of smoothing the cancellous bone intensity. The purpose of smoothing is to decrease the noise generated during the adaptive clustering segmentation. This paper concerns pre-processing of X-ray images of the hand for bone age assessment, and focuses on algorithms and performance of segmentation on hand anatomical structure. Further studies are needed to assess the clinical performance of the method for bone age assessment.

The remainder of this paper is organized as follows: In section background, an overview of the different pre-processing steps in previous literature is discussed. In section methodology, there is an elucidation about the details of the proposed Bounded Area Elimination (BAE). In section result, a number of experiments are carried out: To illustrate the need and effect of anisotropic diffusion as pre-processing; to empirically evaluate the BAE methods output. Finally, conclusions and future directions for research in automated Bone Age Assessment CAD system are discussed in last section.

## Background

A substantial works have been conducted to study the pre-processing of hand skeletal bone from background and soft-tissue region. Majority of the works involve the application of threshold setting which is considered ineffective in the hand bone segmentation due to the fact that the soft-tissue region contains pixel intensity that similar to spongy bone of the hand skeletal bone. Besides, most of the work, after obtaining the region-of-interest (ROI), implements the active contour model which has inherent weaknesses like high sensitivity towards intensity gradient, high dependency on initiation location and low ability in growing into concavity. Some works implement the statistical analysis to determine the membership of each pixel, whether belong to the bone or the soft-tissue region. Some works combine various techniques segmentation in other field into the hand skeletal bone segmentation. The development of the study has been summarized the following paragraphs:

David J. Michael and Alan C. Nelson [15] in 1989 have designed a CAD system for BAA consists of pre-processing, segmentation and measurement. They have processed the image using the histogram equalization follow by converting the image to binary image and implementing the threshold method of pixel's intensity to remove the background using the model parameters. By using the model parameter, the main drawback is that the problem of overlapping of pixel intensity in bone and background could not be resolved; furthermore it is sensitive in illumination change and also the 'shadow' of soft-tissue region around the hand bone. Manos et al. [16] discuss the design of the method for the automatic hand-wrist segmentation. A technique of region growing and region merging after performing the edge detection is implemented during the pre-processing. During the process, threshold is used to determine the edge. Besides, region growing result rely heavily on the initial step where the edge detection is performed. Furthermore, the result of edge detection is uncertain and threshold is involved. The region merging depends on grey level similarity size and connectivity which might combine the epiphysis site that near to the metaphysis.

A group of well-known BAA researchers, Pietka et al. [17] has conducted a study on carpal bone analysis. During the process, thresholding and dilation technique are used for the carpal bones extraction. The algorithm discussed involves dilation that might ruin the result when carpal bones are near with each other. In the following year, Pietka et al. [18] has started to extensively focus on the pre-processing procedure on the bone segmentation from the background using windowing technique to compute the local statistical properties followed by finding the centroid from each peak of the histogram of local window. However, the method does not solve the problem of segmentation with high reliability. The number of peak found in each local window can be uncertain. Errors of computing would occur in some part of the image. In the same year, Sharif et al. [19] have published a paper on bone edge detection Segmentation of bone employing edge detection base on the intensity by the derivative of Gaussian (Drog) followed by the employment of thresholding technique. The pre-processing technique implemented in [20] involve changing the image into binary and performing the thresholding method using histogram to obtain the ROI, the further segmentation of epiphysis within the ROI is implemented through the technique of active shape model. Similarly, the drawbacks of the method are the sensitivity in illumination change and the soft-tissue region. The pre-processing method used in [21] is segmentation of bone using active shape models and a hierarchical bone localization scheme. The method background removing process is performed only after obtaining the ROI.

Mahmoodi et al. [22] carry out binary thresholding to obtain the outline of the hand, followed by location searching of concave-convex; finally the segmentation is performed by the method of active shape models. Pietka et al [23], has conducted a study on image pre-processing and Epiphyseal/Metaphysical ROI Extraction in BAA automated system. The method proposed is about performing the windowing technique and employ the method of adaptive thresholding. The statistical value of mean and variance of each window is then computed to determine the ROI utilizing the technique of star-shaped median filter and Lee filtering to segment the bone from soft-tissue region after obtaining the ROI. Sebastian et al.[24] work on Segmenting the carpal bones from CT images using deformable models, the pre-processing combines the strength of all popular segmentation technique like active contour models, region growing and the global competition in seeded region growing and also the local competition in region competition. The result is satisfying but it involves complicated and heavy computing consumption while computing the partial differential equation. Active contour model [25] has been used in segmenting the bone, the methods [12] c-means clustering algorithm, Gibbs random fields and estimation of the intensity function have been proposed by Pietka et al. They also proposed [26] segmentation of hand bone during pre-processing using the analysis on histogram. By inspecting the peak of the histogram, the authors identify the soft-tissue region and the background.

Gertych et al. [27] use adaptive segmentation method incorporated with Gibbs random field during the pre-processing. Zhang et al. [14] suggest segmenting the carpal by anisotropic diffusion as pre-processing follow by adaptive image threshold setting, binary image labelling and small object removal. However, it involves threshold setting and canny edge detection which are not robust in segmentation. Han et al. [28] propose to implement watershed transform and Gradient vector flow(GVF) to perform the segmentation where the performance of watershed transform and GVF depends heavily on edge gradient strength. Liu et al. [29] implement only primitive image processing technique like edge detection and template matching on the pre-processing segmentation. Giordano et al. [30] perform the segmentation utilizing the derivative difference of Gaussian (DrDog) techniques followed by thresholding using mean and standard deviation.

## Methods

**Figure 1:**
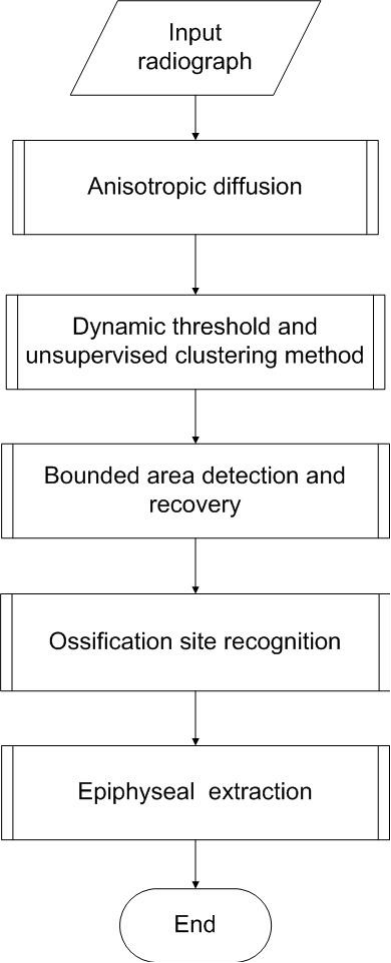
**Dynamic threshold and unsupervised clustering method**. In this figure, a framework of automated Bone Age Assessment (BAA) system is presented. The input radiograph will be diffused by using nonlinear anisotropic diffusion to smooth the image and enhance the edge, preparing it to the segmentation of hand bone from soft-tissue region using adaptive threshold and unsupervised clustering method, followed by a bounded area evaluation to eliminate noise and fill in lost detail; eventually ossification site is recognised, epiphyseal is extracted to be analyzed and bone age is determined.

The automated CAD BAA system begins with a pre-processing with anisotropic diffusion to smooth the non-uniformity within the bone and soft tissue. The image is then processed by adaptive clustering method [13]. The output of the system is then processed by the proposed BAE algorithm to recover the information lost and discard the unwanted information. After obtaining the ROI, the epiphyseal will be extracted. The block diagram of the system processes is depicted in Figure 1

### Image pre-processing using anisotropic diffusion

Prior to most of the image processing techniques such as segmentation and pattern recognition, a filtering process is expected to produce an output image with lower level of noise. There is, however, an inherent problem with the conventional linear filtering like Gaussian filtering: as the noise is being smoothed, the boundaries are smoothed along as well. The first condition is desirable; the second is problematic. To surmount this drawback, the notion of non-linear anisotropic diffusion method based on partial differential equation, proposed by Perona and Malik [31], namely Perona-Malik Anisotropic Diffusion (PMAD), constructed on the basis of scale-space filtering [32] has become a well-known non-linear filtering algorithm for image smoothing. Conventionally, noises are removed by the diffusion algorithms by implementing the heat equation or the isotropic diffusion equation as follows[33]:

(1) ∂I(x,y,t)∕∂t=div(∇I)

Suppose the *I*(*x*, *y*, *t*) denotes the input image at t stage in the continuous domain, where ∇*I*denotes image gradient, *I*(*x*, *y*, 0): *R*^2^→*R*^+^, (*x*, *y*) depicts the spatial position in the image, t depicts the time parameter. The improved version of isotropic partial diffusion equation by Perona and Malik is as follows:

(2) $$\nabla I\left(x,y,t\right)∕\partial t=div\left(g\left(∥\nabla I∥\right)\nabla I\right)$$

Where ||∇*I*|| denotes gradient magnitude, and g (||∇*I*||)denotes the diffusion strength function. The diffusion function controls the intensity of diffusion depends on the image gradient. What makes this anisotropic diffusion having an edge over the conventional scale-space filtering is the existence of the diffusion function -- the edge-preserving or diffusion intensity varying function. This function varies depending on the image gradient: if the magnitude of gradient is large, the intensity of diffusion is low; if the magnitude of gradient is small, the intensity of diffusion is high. This is to fulfil the two final objectives of the image smoothing: the areas within a region are smoothed; boundaries of object (edge) are preserved to keep the edge of object sharp and hence retain the details of the image. To satisfy this characteristic of the diffusion function, two monotonically decreasing diffusion functions have been proposed by Perona and Malik as follows (two dimensions image):

(3) $$g_{1}\left(∥\nabla I∥\right)=\exp\left(-{\left(\frac{∥\nabla I\left(x,y,t\right)∥}{\kappa }\right)}^{2}\right)$$

(4) $$g_{2}\left(∥\nabla I∥\right)=\frac{1}{1+{\left(\frac{∥\nabla I\left(x,y,t\right)∥}{\kappa }\right)}^{1+\alpha }},\alpha >0$$

Where *κ *is a constant, set for adjusting the 'definition of edge'. This value is normally determined by the noise level of the image and the intensity of the edges in image. It is significant for diffusion function to recognize the edges and thus diffusion operation is diminished on them. With the intent to smooth the surface of the bone structure and facilitate the subsequent processing of segmentation, especially segmentation involves clustering; the image underwent anisotropic diffusion with the following algorithm using 2D discrete implementation[34]:

(5) $$\begin{array}{l} gathered\frac{•}{•t}I(x,y,z,t)\\ =div[g(x,y,z,t)*I(x,y,z,t)]\\ =\frac{1}{{\left(\Delta x\right)}^{2}}\\ \left[g(x+\frac{\Delta x}{2}),y,z,t)•(I(x+\Delta x,y,z,t)-I(x,y,z,t)\right)\\ +g(x-\frac{\Delta x}{2},y,z,t)•(I(x-\Delta x,y,z,t)-I(x,y,z,t))]\\ +\frac{1}{{\left(\Delta y\right)}^{2}}\\ \left[g(y+\frac{\Delta y}{2},x,z,t)•(I(y+\Delta y,x,z,t)-I(x,y,z,t)\right)\\ +g(y-\frac{\Delta y}{2},x,z,t)•(I(y-\Delta y,x,z,t)-I(x,y,z,t))]\\ +\frac{1}{{\left(\Delta d_{1}\right)}^{2}}\\ \left[g(x-\frac{\Delta x}{2},y+\frac{\Delta y}{2},z,t)•(I(x-\Delta x,y-\Delta y,z,t)-I(x,y,z,t)\right)\\ +g(x+\frac{\Delta x}{2},y-\frac{\Delta y}{2},z,t)•(I(x+\Delta x,y-\Delta y,z,t)-I(x,y,z,t))]\\ +\frac{1}{{\left(\Delta d_{1}\right)}^{2}}\\ \left[g(x-\frac{\Delta x}{2},y-\frac{\Delta y}{2},z,t)•(I(x-\Delta x,y-\Delta y,z,t)-I(x,y,t)\right)\\ +g(x+\frac{\Delta x}{2},y+\frac{\Delta y}{2},z,t)•(I(x+\Delta x,y+\Delta y,z,t)-I(x,y,z,t))]\\ =\Phi_{east}+\Phi_{west}+\Phi_{north}+\Phi_{south}\\ +\Phi_{eastnorth}+\Phi_{westsouth}+\Phi_{westnorth}+\Phi_{eastsouth} \end{array}$$

For the relative distance, Δx = Δy = 1, Δd = $\sqrt{2}$.

The anisotropic diffusion filtering entails iterative update on each pixel in the image by the flow intensity contributed by its eight neighboring pixels:

(6) $$\begin{array}{l} \frac{•}{•t}I(x,y,t+\Delta t)\\ \approx I(x,y,t)+\Delta t\\ •[\Phi_{east}+\Phi_{west}+\Phi_{north}+\Phi_{south}\\ +\frac{1}{{\left(\Delta d\right)}^{2}}(\Phi_{eastnorth}+\Phi_{westsouth}+\Phi_{{}_{eastsouth})]})]\\ \approx I(x,y,t)+\Delta t\\ •[\Phi_{east}+\Phi_{west}+\Phi_{north}+\Phi_{south}\\ +\frac{1}{2}(\Phi_{eastnorth}+\Phi_{westsouth}+\Phi_{westnorth}+\Phi_{eastsouth})] \end{array}$$

The value of parameter used in pre-processing:

(7) $$g_{2}\left(∥\nabla I∥\right)=\frac{1}{1+{\left(\frac{∥\nabla I\left(x,y,t\right)∥}{\kappa }\right)}^{1+\alpha }}$$

Where *g*_2 _(||∇*I*||)denotes diffusion function and *α *> 0.

Gerig [34] has made an analysis on the diffusion filter integration constant, Δt, and concludes that in 2d discrete implementation of 8 neighboring pixels, the constant range should be in between 0 and 1/7 to ensure the stability. The more Δt is to zero, the better the integration approximates the continuous case. Nevertheless, more iteration are needed by the filter to diffuse the image to a certain extend. The value of the constant is set empirically as 1/7 and iteration is set as 12 in our implementation of the diffusion. The diffusion constant, κ can be viewed as a threshold in determining whether a gradient value is to be smoothed or preserved. If κ is set high, it will become a smoothing filter, where a large gradient might not be treated as edge and therefore is smoothed. On the other hand, if it is set relatively low, the diffusion process will be triggered even in region of high homogeneity. In this paper, the value is set empirically as 12.

### The advantages and disadvantages of anisotropic diffusion

**Figure 2:**
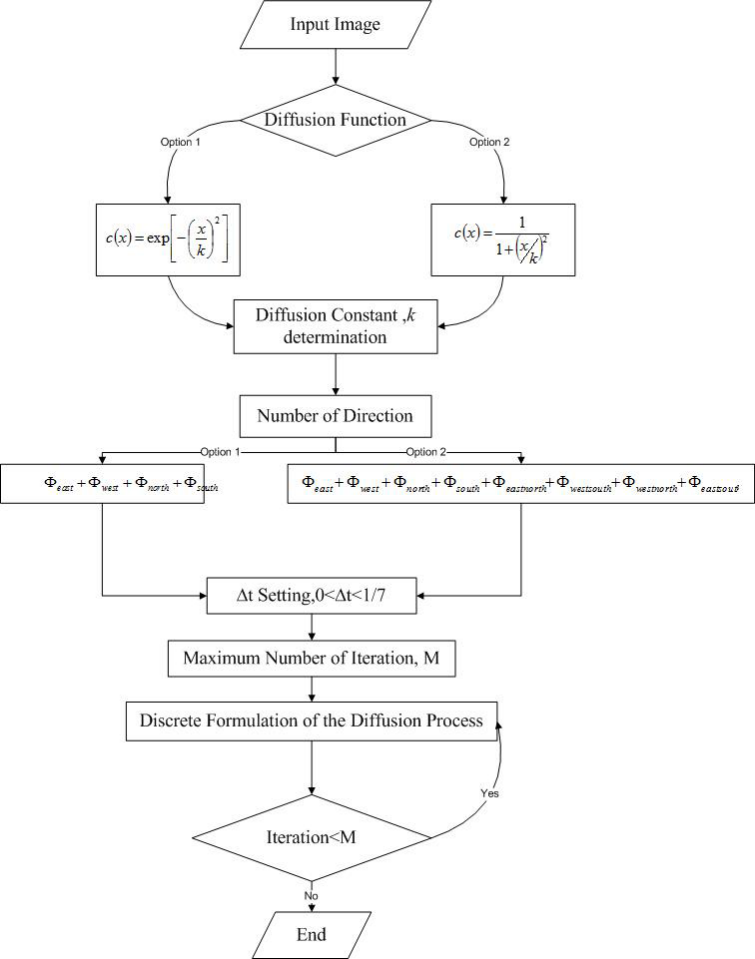
**Flow chart for two dimensional anisotropic diffusion**. In this figure, the flow of anisotropic diffusion is presented. A diffusion function has to be chosen, followed by determining a constant, *κ*. This constant is essential to define the edges and homogenous area; hence, different diffusion intensity is imposed on them. Subsequently, number of directions is to be chosen: either four directions or eight directions, as common practice. Then, the choice of integration constant, Δ*t*: the effect of Δ*t *can be viewed as an approximation for continuous case in integration for discrete implementation. Eventually, stopping criterion is to be determined: the maximum number of iteration.

In comparison to the conventional scale-space filtering methods,[35] anisotropic diffusion possesses distinct major advantages: the relatively low computational complexity of anisotropic diffusion increases its applicability for general purposes; anisotropic diffusion takes objects' edges into account during filtering; hence, the edge is not blurred and the details are preserved. Boundaries of objects, hence, are sharpened and can be clearly defined. Besides, the anisotropic diffusion is capable of manipulating the intensity of diffusion direction to assure no cross diffusion occurs at edges;[36] but assure occurrence of diffusion in direction parallel with edges; thus, not only edges are preserved, the edges are enhanced. This is crucial in medical image processing where organ or tumors contours must able to be distinguished clearly. Despite having these advantages, anisotropic diffusion contains limitations: the method requires value setting of constants such as *κ*: to maximize the edge preserving and noise filtering purposes of anisotropic diffusion, the constants have to be optimally tuned; if constant is not selected correctly, undesirable effect would occur: small continuities among tissues in medical imaging would be blurred and noises are considered as edges and hence the noises are intensified. It is claimed that [37] the anisotropic diffusion proposed by Perona and Malik do not incorporate the convergence criterion and difficult to determine when to halt the iteration process. The flow chart of anisotropic diffusion is presented in Figure 2.

### Post-processing of surrounded area restoration

After the implementation dynamic threshold segmentation technique, due to the nature of the pixels intensity distribution in hand bond, there are areas especially in the regions of cancellous bone in the finger, would be segmented as well. This phenomenon has led to the problem where the bone area is segmented, which is not desirable. In this paper, a method called Bounded Area Evaluation (BAE) is proposed.

The motivation of BAE can be analyzed from two points of view: the relationship between feature extraction and classification, and the inherent drawbacks of segmentation based on adaptive thresholding. Feature extraction is performed between segmentation and ossification site localization: for instance, segmented bone radiograph will undergo feature extraction; features such as anatomical structure boundary such as edge, number of concavities, and curvature; regional properties such as bone area and perimeter, statistical information such as mean, standard deviation, and kurtosis; characteristic function such as invariants moments of bone, texture information such as entropy, uniformity, and pixel's neighborhood relationship. Type of features employed depends on the classifier in latter stage during ossification center localization (pattern recognition and object detection).

The relationship [38] of extracted features and classifier is complementary: for sophisticated features extraction, a simple classifier is sufficient to perform the pattern recognition; conversely, for unsophisticated features extraction, a supreme classifier is required to sufficiently perform the pattern recognition. Therefore, a segmented bone, without noises and loss of detail is vital in assuring features can be extracted and subsequently accurate pattern recognition can be performed. The proposed BAE algorithm eliminates the noises and fills in the lost details after adaptive segmentation. Adaptive segmentation is more robust than global thresholding segmentation; however, it has an inherent limitation where resultant images are always defected by various noises and loss of details. The image artifacts produced will affect the abovementioned feature extraction process and result in inaccurate pattern recognition in bone age assessment system. It is, therefore, the main objective of the proposed BAE algorithm is to compensate the segmentation defects by detecting the bounded area outside the bone area, and replace it with background pixel intensity and by detecting the bounded area inside the bone area and replace it by the original bone pixel intensity.

### Bounded Area Evaluation (BAE) algorithm

**Figure 3:**
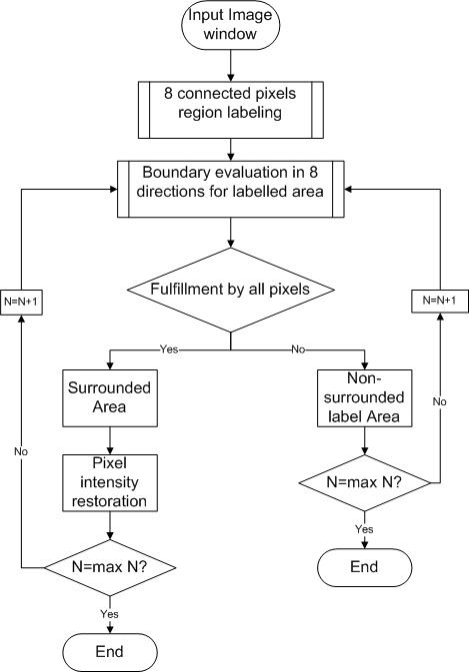
**Flow chart for the bounded area evaluation (BAE) algorithms**. In this figure, the main structure of BAE algorithms is illustrated. The input image will undergo a region labeling process of eight connected pixels. After that an evaluation of boundary of each label cluster is performed. Two errors are expected to be found: the surrounded area represents the lost detail; the non-surrounded label represents noises and redundant information. The undesired noise will be eliminated while the lost detail will be recovered.

**Input**: Data set (image pixels with label) = $f\left(x,\;y\right)I_{x,y}^{n}$ where 'n' represents the number order of label, 'x' and 'y' represent the coordinate of the corresponding pixel; f(x, y) denotes the switching function. The input image for the BAE is labeled image using the procedure described in [1] using the 8 connected object, after the image is labeled, each member for each labeled cluster will undergo a testing procedure to ensure each pixel in each direction of a certain labeled cluster fulfill the requirement. The flow chart of BAE is illustrated in Figure 3 and the process of BAE is mathematically defined in table 1.

**Table 1:** The BAE algorithm

Process	Equation
(a)Evaluate each pixel in n-label for 0 degree	$f\left(x,y\right)=\left\{\begin{array}{c} 0,I_{x+k,y}^{b}\;\text{if}\;\text{b = n,}\\ 1,I_{x+k,y}^{b}\;\text{if}\;\text{b}\neq \text{n,} \end{array}\right)$ Where k = 1→L L = maximum number of column -x
Evaluate each pixel for 45 degree	$f\left(x,y\right)=\left\{\begin{array}{c} 0,I_{x+m,y-k}^{b}\;\text{if}\;\text{b = n,}\\ 1,I_{x+m,y-k}^{b}\;\text{if}\;\text{b}\neq \text{n,}\text{.} \end{array}\right)$ Where k = 1→y - 1, m = 1→L L = maximum number of column -x
Evaluate each pixel in n-label for 90 degree	$f\left(x,y\right)=\left\{\begin{array}{c} 0,I_{x,y-k}^{b}\;\text{if}\;\text{b = n,}\\ 1,I_{x,y-k}^{b}\;\text{if}\;\text{b}\neq \text{n,} \end{array}\right)$ Where k = 1→y - 1
Evaluate each pixel in n-label for 135 degree	$f\left(x,y\right)=\left\{\begin{array}{c} 0,I_{x-m,y-k}^{b}\;\text{if}\;\text{b = n,}\\ 1,I_{x-m,y-k}^{b}\;\text{if}\;\text{b}\neq \text{n,} \end{array}\right)$ Where k = 1→y - 1, m = 1→x - 1
Evaluate each pixel in n-label for 180 degree	$f\left(x,y\right)=\left\{\begin{array}{c} 0,I_{x-m,y}^{b}\;\text{if}\;\text{b = n,}\\ 1,I_{x-m,v}^{b}\;\text{if}\;\text{b}\neq \text{n,} \end{array}\right)$ Where k = 1→y - 1, m = 1→x - 1
Evaluate each pixel in n-label for 225 degree	$f\left(x,y\right)=\left\{\begin{array}{c} 0,I_{x-m,y+k}^{b}\;\text{if}\;\text{b = n,}\\ 1,I_{x-m,y+k}^{b}\;\text{if}\;\text{b}\neq \text{n,} \end{array}\right)$ Where k = 1→L, m = 1→x - 1 L = maximum number of row -x
Evaluate each pixel in n-label for 270 degree	$f\left(x,y\right)=\left\{\begin{array}{c} 0,I_{x,y+k}^{b}\;\text{if}\;\text{b = n,}\\ 1,I_{x,y+k}^{b}\;\text{if}\;\text{b}\neq \text{n,} \end{array}\right)$ Where k = 1→L L = maximum number of row -x
Evaluate each pixel in n-label for 315 degree	$f\left(x,y\right)=\left\{\begin{array}{c} 0,I_{x+m,y+k}^{b}\;\text{if}\;\text{b = n,}\\ 1,I_{x+m,y+k}^{b}\;\text{if}\;\text{b}\neq \text{n,} \end{array}\right)$ Where k = 1→L1, m = 1→L2 L1 = maximum number of row-y L2 = maximum number of column -x
(b)Stopping criteria:	[*f*(*x*, *y*) = 1] ∪ n = N *N *= *maximum label*
(c)Verification of bounded area for n-label	$bounded\;area,\;if\frac{\sum_{x}^{\max\;x}\sum_{y}^{\max\;y}f\left(x,y\right)I_{x,y}^{n}}{\begin{array}{c} total\;number\;of\;pixels\;in\;n-label\\ non-bounded\;area,\;otherwise \end{array}}=1$
(d)Repeat the process	n = n+1, where n denotes the label number of pixels in image.
(e)Fill the pixels belong to bounded area with original value/background value	$I_{x,y}=I_{x,y}^{n}$

This table illustrates the steps in the BAE process. Step in part (a) demonstrates the labelling process in each direction. Step in part (b) explains the stopping criteria. Step in part (c) defines the recognition of bounded area, for it a noise or lost data. The entire process mentioned above is repeated in step in part (d). Last step involves the filling in the lost data or elimination of noise.

## Results

Two categories of experiments based on the result of anisotropic diffusion and the proposed BAE algorithms are set up to serve the purposes as following:

(a) (i) To prove qualitatively that the image of hand radiograph can be smoothed by anisotropic diffusion.

(ii) To prove quantitatively that the variation in pixels intensity in soft-tissue region and bone can be suppressed by anisotropic diffusion.

(iii) Qualitative comparison on anisotropic diffusion with other alternatives to demonstrate the performance of anisotropic diffusion.

(b) (i) To prove qualitatively that the output image of adaptive clustering segmentation for hand is improved by BAE algorithm.

(ii) To prove quantitatively that the 'busyness' of the image has been reduced after the implementation of BAE algorithm.

### Qualitative analysis on the effect of anisotropic diffusion on radiographs

**Figure 4:**
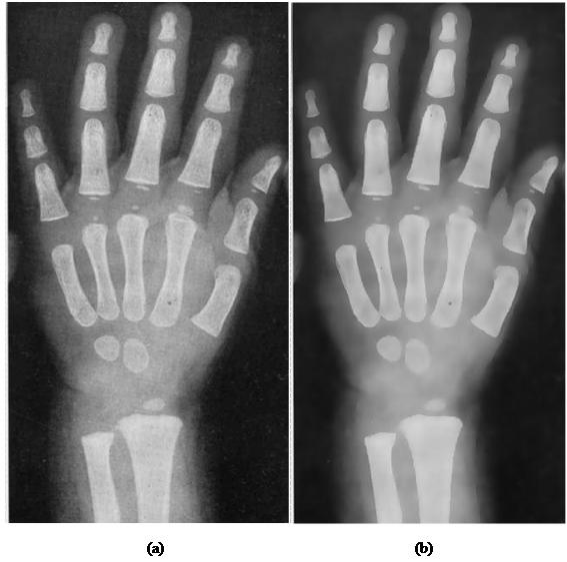
**Comparison of radiographs before and after anisotropic diffusion**. In this figure, a child's left hand bone radiograph is used to demonstrate the anisotropic diffusion effect: (a) Hand radiograph image before diffusion (b) Hand radiograph image after diffusion. As the result presents, the bone area has been smoothed to become homogenous area; the black holes and dots in have been filled by similar pixel intensity with the surrounding bone. Despite this filtering process, the edges of hand structure are preserved and can be clearly seen.

The image before and after diffusion are shown in Figure 4, by visual inspection, it is apparent that the image after anisotropic diffusion is smoothed while still being able to preserve the edge of the bone structure. Besides, the bone intensity has been diffused into a smooth area, where the pixel intensity in the spongy bone area has been equalized to a common level of pixel intensity. This will finally favor the adaptive segmentation in two ways: similar data, in this case -- the bone area, possess more similar intensity level; the spongy bone becomes more distinguishable to the soft-tissue region.

### Quantitative analysis of anisotropic diffusion on hand radiograph

In Figure 4, it is apparent that the high variation pixels within the hand bone and soft-tissue region is smoothed while the edge of the anatomical structure remains sharp. In other words, the smoothing mechanism is within boundaries and it is desired for subsequent process. It is the uniqueness of anisotropic diffusion that it promotes intra-region smoothing rather than inter-region smoothing. The anisotropic diffusion is applied on 100 images randomly chosen from digital atlas database from different age groups and races to assess quantitatively the impacts imposed on image through unsupervised texture evaluation[39]. Homogeneity (64 gray levels) and variance are chosen as the measurement index in the assessment. Table 2 presents the result obtained. The equations used in computing the result are as following.

**Table 2:** Comparison of image homogeneity of different age group before and after the anisotropic diffusion processing

Age Group	Image Homogeneity Before Anisotropic Diffusion	Image Homogeneity After Anisotropic Diffusion
0-3	0.7056	**0.8235**
3-6	0.6984	**0.8245**
7-9	0.7132	**0.8365**
10-12	0.7189	**0.8423**
12-14	0.7028	**0.8212**
14-16	0.6927	**0.8234**
16-18	0.7056	**0.8345**

This table tabulates the homogeneity value of hand radiograph before and after the anisotropic diffusion. 100 test images are chosen: the test image chosen randomly from each group age of children with different shapes and sizes. The homogeneity is based on the gray level co-occurrence matrix; it illustrates the texture of the resultant image: higher value indicates higher degree of smoothness and vice versa.

(8) $$\text{Contrast}=\frac{1}{8}\sum_{i,j=0}^{N-1}p_{i,j}{\left(i-j\right)}^{2}$$

(9) $$\text{Homogeneity}\left(\Theta \right)=\frac{1}{8}\sum_{i,j=0}^{N-1}\frac{p_{i,j}}{1+{\left(i-j\right)}^{2}}$$

Where *P_i, j _*denotes the probability of occurrence a group of spatial related pixel intensity in a unit distance and θ direction. Eight directions chosen in this experiment are: 45°, 90°, 135°, 180°, 225°, 270°, 315°, 360°. N denotes the maximum number of gray level implemented in the calculation. The N chosen in this experiment is 64.

Note that homogeneity, or 'inverse difference moment' is an inversion to the contrast. The only difference is that the weight for the element proportional to the distance away from diagonal: while computing the contrast, the weight of element increases as the distance of element from diagonal of the gray level co-occurrence matrix [40] increases. Inversely, [13,41,42] the weight of element decreases as the distance of elements from diagonal increases. In short, the weight of contrast and homogeneity are (*i *- *j*)^2 ^and $\frac{1}{1+{\left(i-j\right)}^{2}}$respectively. Therefore, to avoid redundancy, the texture analysis is computed using only homogeneity. Table 2 compared the smoothness of image after and before the implementation of anisotropic diffusion in different age group. The higher value will be bold to ease the comparison. The result indicates that the image homogeneity after anisotropic diffusion is improved averagely on each age group for 17.59%.

### Qualitative analysis on anisotropic diffusion with other alternatives

**Figure 5:**
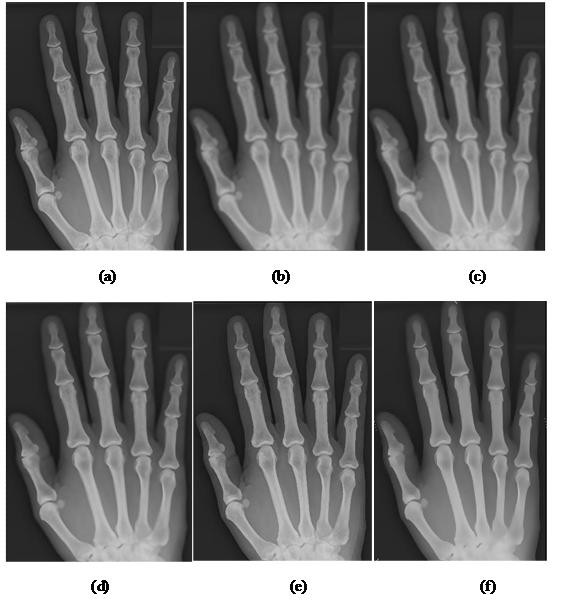
**Comparisons of radiograph processed by anisotropic diffusion with various alternatives**. In this figure, the radiograph is diffused by different algorithm for comparison: **(**a) Original image (b) Gaussian filter (c) Average filter (d) Wiener filter (e) Symmetric Nearest Neighbor (SNN) filter (f) Anisotropic diffusion.

100 of test images from each age group are selected randomly to perform the qualitative analysis test; the result is consistent; only one is shown in Figure 5 for illustration. From the result in Figure 5, it is found that the Gaussian filter [43] and average filter[44] produce filtered image with blurred edges; Wiener filter[45] produces better diffused image but it is not satisfying at some spots of the image and the improvement is not obvious in spongy bone area; Symmetric Nearest Neighbor (SNN) filter [46] produces a sharpen edge image but the intensity within bone structures are not diffused; anisotropic diffusion produces an edge-preserving and a satisfied diffusion effect on the resultant image.

### Quantitative analysis on the BAE algorithms

Qualitative evaluation by human visual system is subjective and the evaluation varies depending on the observer's perspective and background. Therefore, quantitative analysis on the segmented image is crucial to compare the relative effectiveness among segmentation methods. However, computing a quantitative score that can objectively and accurately reflect the performance of segmentation has been a daunting task. There are two main types of quantitative evaluation [39]: supervised evaluation and unsupervised evaluation. Supervised evaluation entails comparison between segmented image and a reference image; unsupervised evaluation entails no reference image in the process of evaluation. The reference image is acquired by either manually segmentation or pre-processed ground truth image which involves drawbacks like subjective human visual perspective, tedious, time-consuming. In the contrary, no ground truth image is required to perform the unsupervised evaluation, and therefore, it is more objective and feasible in comparing segmented objects' structures. Therefore, in this paper, we adopt the recently proposed objective unsupervised evaluation [47] -- Mean Structural SIMilarity (MSSIM) index. This index has been proven [48] to be more robust (consider more properties and correspond to perspective of human) than the conventional image quality metrics evaluation methods [49] such as Mean Square Error (MSE), Peak Signal-to-Noise Ratio(PSNR)and Entropy.

The SSIM consists of three components: luminance function, contrast function, and structure function. The definition of luminance function is as follow:

(10) $$l\left(X,Y\right)=\frac{2\mu_{X}\mu_{Y}+C_{1}}{\mu_{X}^{2}+\mu_{Y}^{2}+C_{1}}$$

Where *X *depicts input image, *Y *depicts output image, *μ_X _*depicts expected value(mean) of input image, *μ_Y _*denotes expected value of output image, *C*_1 _denotes constant. The function *l*(*X*, *Y*) illustrates a luminance comparison metric where the constant *C*_1 _to stabilize the output of function in extreme case -- both the mean of input image and output image close to zero. The maximum value of *l*(*X*, *Y*) equals to one only if both input image and resultant image have identical mean. As the relative mean difference between input image and resultant image increases, the function approaches zero. Similarly, the contrast function is represented mathematically as follows:

(11) $$c\left(X,Y\right)=\frac{2\sigma_{X}\sigma_{Y}+C_{2}}{\sigma_{X}^{2}+\sigma_{Y}^{2}+C_{2}}$$

Where *σ_X _*represents standard deviation of input image, *σ_Y _*represents standard deviation of output image, *C*_2 _is a constant. The function *c*(*X*, *Y*) depicts a contrast comparison metric where the constant *C*_2 _with the purpose of stabilizing the function. This function has bounded value of one (maximum), occurs if both input and output images generate identical standard deviation. The third component, structural comparison function, is defined as follows:

(12) $$s\left(X,Y\right)=\frac{\sigma_{XY}+C_{3}}{\sigma_{X}\sigma_{Y}+C_{3}}$$

Where *σ_XY _*depicts covariance of input image and resultant image defined as follows:

(13) $$\sigma_{XY}\left(X,Y\right)=\frac{1}{\left(N-1\right)}\overset{N}{\underset{i=1}{\sum }}\left(X_{i}-\mu_{X}\right)\left(Y_{i}-\mu_{Y}\right)$$

The covariance describes the structure (contour and outline of objects) in the image -- the detail of the image. Covariance compares the changes of intensity in respective pixel in image: if a particular pixel of input image has pixel intensity lower than the input image expected value, and this relative relationship remains in resultant image, a positive value proportional to the difference will contribute in the function output; if the intensity value of a pixel in input image is more than the mean value, and this condition hold for output image, then the covariance of the particular pixel between the input image and output image will as well be a positive value. Positive value, of covariance, therefore, could describe the deviation of structure in the segmented image compare to the original image. On the contrary, if the relationship between a particular pixel between input and output image is varying inversely, it will lead to a contribution of negative value in covariance and thus negative covariance describes a structural change during an image processing. The covariance is then normalized by the multiplication of *σ_X _*and *σ_Y _*so that if two images are identical, the value of structure comparison will become unity assuming that *C*_3 _is a relatively small constant.

Finally, the three functions are combined to become the SSIM between the input image, *X *and output image, *Y *as follows:

(14) $$SSIM\left(X,Y\right)={\left[l\left(X,Y\right)\right]}^{\alpha }.{\left[c\left(X,Y\right)\right]}^{\beta }.{\left[s\left(X,Y\right)\right]}^{\gamma }$$

Where *α *> 0, *β *> 0, *γ *> 0. Adjusting the parameter manipulates the relative importance of each function in SSIM. Note that the constants, *C*_1 _, *C*_2 _and *C*_3 _are defined as *C_i _*= (*K_i _L*)^2 ^Where *K_i _*≪ 1 for *i *= 1, 2, 3. Note that the parameters used in this paper are *α *= 1, *β *= 1, *γ *= 1, or in other words, in this paper, we consider the components are all equally important, and *K*_1 _= 0.01, *K*_2 _= 0.03,$C_{3}=\frac{C_{2}}{2}$. It is proven [48] that the value of constant is insensitive to the SSIM as long as it is far less than one. Besides, in this paper, for illustration purpose, global SSIM will be performed rather than local SSIM. Note that the parameters used in this paper are *α *= 1, *β *= 1, *γ *= 1, we consider the components are all equally important, and *K*_1 _= 0.001, *K*_2 _= 0.001, *K*_3 _= 0.001. The local statistics are computed using an 11 × 11 circular symmetric Gaussian weighting function with standard deviation 1.5, normalized to unit sum as suggested in [48]. The obtained values for each local window are divided by the number of local windows in the image as Mean SSIM (MSSIM) as follow:

(15) $$MSSIM\left(X,Y\right)=\frac{1}{M}\overset{M}{\underset{j=1}{\sum }}SSIM\left(x_{j},y_{j}\right)$$

Where *X *denotes input image; *Y *denotes resultant image; *x_j _*and *y_j _*denote image pixels in the *j *- *th *local window respectively; and M denotes the total number of local windows. In addition, for better assessment, homogeneity is also adopted to gauge the texture of the hand bone radiograph after diffusion filtering and the texture of the hand bone radiograph after segmentation using resultant image that have undergone the BAE algorithm.

The BAE algorithm has been implemented on the adaptive clustering segmentation algorithm[13]. The number of row tested are from 2 to 19, number of column of each row is from 2 to 10 and the result is evaluated using the smoothness metric, homogeneity to assess the 'busyness' of the radiograph before and after the BAE algorithms. Results of experiments showed that the smoothness has been improved averagely 35% in table 3. From table 4, the MSSIM is improved averagely 10.49% after performing the BAE algorithm. This indicates that the lost of detail and structural changes in segmented image is lower for images that have undergone pre-processing by BAE algorithm.

**Table 3:** Comparison of homogeneity value before and after the BAE algorithms processing

Bands		Homogeneity		Bands		Homogeneity	
			
Row	Column	Before	After	Row	Column	Before	After
2	2-10	0.6508	0.8815	11	2-10	0.6380	0.8814
3	2-10	0.6657	0.8820	12	2-10	0.6471	0.8812
4	2-10	0.6672	0.8811	13	2-10	0.6698	0.8905
5	2-10	0.6412	0.8830	14	2-10	0.6503	0.8821
6	2-10	0.6671	0.8806	15	2-10	0.6661	0.8903
7	2-10	0.6655	0.8806	16	2-10	0.6629	0.9012
8	2-10	0.6309	0.8803	17	2-10	0.6547	0.8805
9	2-10	0.6402	0.8833	18	2-10	0.6663	0.8907
10	2-10	0.6589	0.8827	19	2-10	0.6652	0.9110

The resultant images (randomly from each age group) of Adaptive Clustering Reconstruction (ACR) segmentation with different row and column number are tested with BAE algorithms. The average homogeneity of each resultant image before and after performing BAE algorithms is tabulated: The result shows that the homogeneity is higher after BAE algorithms.

**Table 4:** Mean Structural Similarity (MSSIM) before and after the BAE algorithm processing

Bands		MSSIM		Bands		MSSIM	
			
Row	Column	Before	After	Row	Column	Before	After
2	2-10	0.8345	0.9324	11	2-10	0.8640	0.9223
3	2-10	0.8423	0.9431	12	2-10	0.8595	0.9343
4	2-10	0.8355	0.9341	13	2-10	0.8420	0.9243
5	2-10	0.8785	0.9125	14	2-10	0.8298	0.9089
6	2-10	0.8523	0.9256	15	2-10	0.8482	0.9423
7	2-10	0.8397	0.9543	16	2-10	0.8210	0.9432
8	2-10	0.8450	0.9354	17	2-10	0.8323	0.9502
9	2-10	0.8574	0.9445	18	2-10	0.8489	0.9357
10	2-10	0.8259	0.9213	19	2-10	0.8518	0.9389

This table compares the MSSIM of each image tested in previous table 3 to evaluate the structural preserving ability of BAE. The result shows that the MSSIM before BAE algorithm is consistently lower than the MSSIM after performing BAE algorithm. This indicates that the BAE algorithm has contributed in preserving detail of the image. The MSSIM is improved averagely 10.49% after performing the BAE algorithm on the adaptive segmented hand radiograph.

### Qualitative analysis of BAE algorithm

**Figure 6:**
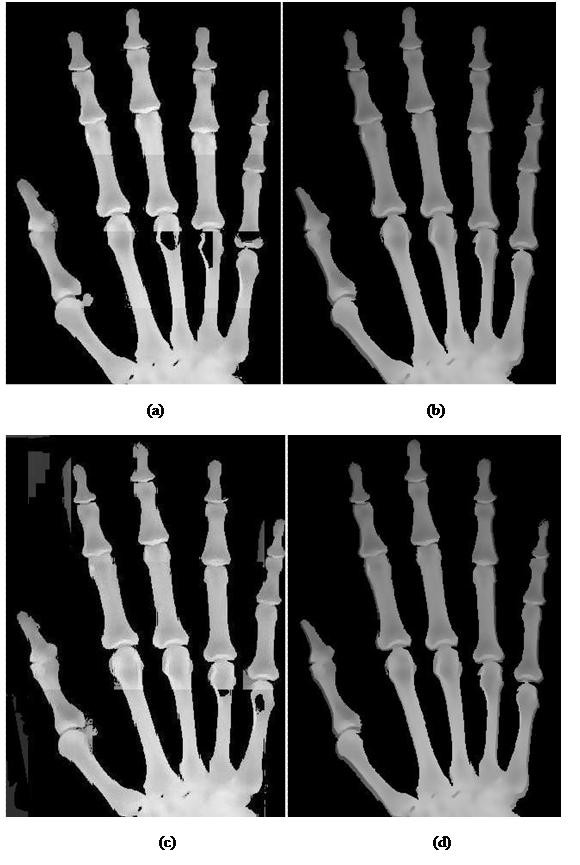
**Comparison of hand radiograph before and after the BAE algorithms**. In this figure, the image presented are (a) Hand radiograph before BAE (b) Recovery of the artifacts of finger radiograph (c) Hand radiograph with anomalies and noise (d) Finger radiograph free from anomalies and noise after BAE.

The image before the BAE algorithm confronts with two problems: the occurrence of anomalies and incorrectly segmented bone regions. The result before and after BAE algorithm is presented in Figure 6. From the radiograph presented in Figure 6, it is apparent that the anomalies have been removed and the lost data have been recovered after the implementation of BAE algorithms. For the qualitative analysis, 100 images have been randomly picked from each age group to be qualitatively analyzed. The result shows that the image after BAE algorithm consistently contains less visual artifacts and lost detail have been recovered.

## Conclusions

This paper proposed a new algorithm, namely, bounded area evaluation (BAE) algorithms besides suggesting the implementation of anisotropic diffusion as pre-processing to adaptive segmentation: The nonlinear diffusion technique is implemented to smooth the image and cover the bone area with equalized intensity in order to enhance the latter process of adaptive segmentation. The BAE algorithm is designed as post-processing to eliminate the noise created during clustering segmentation of bone structure. Two types of experiments have been set up to conclude the findings: one is for the anisotropic diffusion competency assessment; the other is for the BAE algorithm performance assessment. The results of the finding are evaluated quantitatively and qualitatively. The conclusion we obtained are: The smoothness of the hand radiograph is improved effectively by the implementation of anisotropic diffusion; the BAE algorithm is able to recover the data lost and remove the anomalies created during the clustering segmentation. Besides, the success in post processing in the development of the segmentation technique can be utilized to resolve the common problems like variation in bone mineralization, the variation in shape of different bone, the existence of soft-tissue region that affect the CAD system's performance in various CAD radiograph diagnosis system and the inconsistency of grey levels between the bone and soft-tissue region. The success of segmentation determines the development of subsequent CAD system in medical diagnosis. The research topic is believed to be capable of sparking more research towards the field of medical imaging analysis. In future works, focus will be put on minimizing the parameters used during the implementation of anisotropic diffusion and BAE. It is recommended that the algorithms would automatically determine the optimized value of parameters during the process of segmentation to obtain the optimized result.

## Competing interests

The authors declare that they have no competing interests.

## Authors' contributions

HYC designed the proposed BAE algorithms for segmentation of hand bones. LKW participated in acquiring data and pre-processing of hand bone segmentation. TTS conducted qualitative analysis both on anisotropic diffusion and BAE processed images. HS conducted quantitative analysis on both anisotropic diffusion and BAE processed images. LYC participated in mathematical analysis on data and coordination of the project. All authors read and approved the final manuscript.

## References

[B1] Cao F Huang HK Pietka E Gilsanz V Digital hand atlas and web-based bone age assessment: system design and implementation Computerized Medical Imaging and Graphics 2000 24 297 307 10.1016/S0895-6111(00)00026-4 10940607

[B2] Roche AF French NY Differences in skeletal maturity levels between the knee and hand Am J Roentgenol 1970 109 307 312 10.2214/ajr.109.2.307 4316030

[B3] Heinrich UE Significance of radiologic skeletal age determination in clinical practice Die Bedeutung der radiologischen Skelettalterbestimmung für die Klinik 1986 26 212 215 3726094

[B4] Peloschek P Nemec S Widhalm P Donner R Birngruber E Thodberg HH Kainberger F Langs G Computational radiology in skeletal radiography European Journal of Radiology 2009 72 252 257 10.1016/j.ejrad.2009.05.053 19581060

[B5] Greulich W Pyle S Radiographic atlas of skeletal development of hand wrist 1959

[B6] Tanner J Whitehouse R Assessment of skeletal maturity and prediction of adult height(TW2 method) 1975

[B7] Acheson RM Fowler G Fry EI Janes M Koski K Urbano P Werfftenboschjj VA Studies in the reliability of assessing skeletal maturity from x-rays. 3. Greulich-Pyle Atlas and Tanner-Whitehouse method contrasted Human biology; an international record of research 1963 35 317 349 14065196

[B8] Ontell FK Ivanovic M Ablin DS Barlow TW Bone age in children of diverse ethnicity American Journal of Roentgenology 1996 167 1395 1398 8956565 10.2214/ajr.167.6.8956565

[B9] Tanner JM Gibbons RD Automatic bone age measurement using computerized image analysis Journal of Pediatric Endocrinology 1994 7 141 145 10.1515/JPEM.1994.7.2.141 8061759

[B10] Cole AJL Webb L Cole TJ Bone age estimation: a comparison of methods Br J Radiol 1988 61 683 686 10.1259/0007-1285-61-728-683 3416108

[B11] Jonsson K Fundamentals of Hand and Wrist Imaging Acta Radiologica 2002 43 236 236

[B12] Pietka E Pospiech-Kurkowska S Gertych A Cao F Integration of computer assisted bone age assessment with clinical PACS Computerized Medical Imaging and Graphics 2003 27 217 228 10.1016/S0895-6111(02)00076-9 12620312

[B13] Chai HY Wee LK Swee TT Salleh SH Adaptive Crossed Reconstructed (ACR) K-mean Clustering Segmentation for Computer-aided Bone Age Assessment System International Journal of Mathematical Models and Methods in Applied Sciences 2011 5

[B14] Zhang A Gertych A Liu BJ Automatic bone age assessment for young children from newborn to 7-year-old using carpal bones Computerized Medical Imaging and Graphics 2007 31 299 310 10.1016/j.compmedimag.2007.02.008 17369018 PMC2041862

[B15] Michael DJ Nelson AC HANDX: A model-based system for automatic segmentation of bones from digital hand radiographs IEEE transactions on medical imaging 1989 8 64 69 10.1109/42.20363 18230501

[B16] Manos G Cairns AY Ricketts IW Sinclair D Automatic segmentation of hand-wrist radiographs Image and Vision Computing 1993 11 100 111 10.1016/0262-8856(93)90076-S

[B17] Pietka E Kaabi L Kuo ML Huang HK Feature extraction in carpal-bone analysis IEEE transactions on medical imaging 1993 12 44 49 10.1109/42.222665 18218390

[B18] Pietka E Computer-assisted bone age assessment based on features automatically extracted from a hand radiograph Computerized Medical Imaging and Graphics 1994 19 251 259 10.1016/0895-6111(95)00005-b 7641169

[B19] Sharif BS Zaroug SA Chester EG Owen JP Lee EJ Bone edge detection in hand radiographic images Baltimore, MD, USA. IEEE 1994 514 515

[B20] Mahmoodi S Sharif BS Chester EG Owen JP Lee REJ Automated vision system for skeletal age assessment using knowledge based techniques Dublin, Irel. IEE 1997 809 813

[B21] Mahmoodi S Sharif BS Chester EG Owen JP Lee REJ Bayesian estimation of growth age using shape and texture descriptors Manchester, UK. IEE 1999 489 493

[B22] Mahmoodi S Sharif BS Graeme Chester E Owen JP Lee R Skeletal growth estimation using radiographie image processing and analysis IEEE Transactions on Information Technology in Biomedicine 2000 4 292 297 10.1109/4233.897061 11206814

[B23] Pietka E Gertych A Pospiech S Cao F Huang HK Gilsanz V Computer-assisted bone age assessment: Image preprocessing and epiphyseal/metaphyseal ROI extraction IEEE transactions on medical imaging 2001 20 715 729 10.1109/42.938240 11513023

[B24] Sebastian TB Tek H Crisco JJ Kimia BB Segmentation of carpal bones from CT images using skeletally coupled deformable models Medical Image Analysis 2003 7 21 45 10.1016/S1361-8415(02)00065-8 12467720

[B25] Sotoca JM Iñesta JM Belmonte MA Hand bone segmentation in radioabsorptiometry images for computerised bone mass assessment Computerized Medical Imaging and Graphics 2003 27 459 467 10.1016/S0895-6111(03)00053-3 14575779

[B26] Pietka E Gertych A Pospiechâ€"Kurkowska S Cao F Huang HK Gilzanz V Computer-Assisted Bone Age Assessment: Graphical User Interface for Image Processing and Comparison Journal of Digital Imaging 2004 17 175 188 10.1007/s10278-004-1006-6 15175931 PMC3046610

[B27] Gertych A Piętka E Liu B Segmentation of regions of interest and post-segmentation edge location improvement in computer-aided bone age assessment Pattern Analysis & Applications 2007 10 115 123 10.1007/s10044-006-0056-421966567

[B28] Han C-C Lee C-H Peng W-L Hand radiograph image segmentation using a coarse-to-fine strategy Pattern Recognition 2007 40 2994 3004 10.1016/j.patcog.2007.01.010

[B29] Liu J Qi J Liu Z Ning Q Luo X Automatic bone age assessment based on intelligent algorithms and comparison with TW3 method Computerized Medical Imaging and Graphics 2008 32 678 684 10.1016/j.compmedimag.2008.08.005 18835130

[B30] Giordano D Spampinato C Scarciofalo G Leonardi R An automatic system for skeletal bone age measurement by robust processing of carpal and epiphysial/metaphysial bones IEEE Transactions on Instrumentation and Measurement 2010 59 2539 2553

[B31] Perona P Anisotropic diffusion processes in early vision Multidimensional Signal Processing Workshop, 1989, Sixth; 6-8 Sep 1989 1989 68

[B32] Witkin A Scale-Space Filtering8th Int Joint Conf Artificial Intelligence 1983 1019 1022

[B33] Perona P Malik J Scale-space and edge detection using anisotropic diffusion Pattern Analysis and Machine Intelligence, IEEE Transactions on 1990 12 629 639 10.1109/34.56205

[B34] Gerig G Kubler O Kikinis R Jolesz FA Nonlinear anisotropic filtering of MRI data Medical Imaging, IEEE Transactions on 1992 11 221 232 10.1109/42.141646 18218376

[B35] Black MJ Sapiro G Marimont DH Heeger D Robust anisotropic diffusion Image Processing, IEEE Transactions on 1998 7 421 432 10.1109/83.661192 18276262

[B36] Wang G Sang N Yan L Shen X X-ray angiogram images enhancement by facet-based adaptive anisotropic diffusion Computerized Medical Imaging and Graphics 2009 33 140 147 10.1016/j.compmedimag.2008.11.001 19095408

[B37] Nordstrom KN Biased anisotropic diffusion: a unified regularization and diffusion approach to edge detection Image Vision Comput 1991 8 318 327

[B38] Duda R Hart P Stork D Pattern Classification 2001 2 Wiley-Interscience

[B39] Zhang H Fritts JE Goldman SA Image segmentation evaluation: A survey of unsupervised methods Computer Vision and Image Understanding 2008 110 260 280 10.1016/j.cviu.2007.08.003

[B40] Haralick RM Shanmugam K Dinstein IH Textural Features for Image Classification Systems, Man and Cybernetics, IEEE Transactions on 1973 3 610 621

[B41] Chai HY Wee LK Swee TT Salleh SH Ariff AK Kamarulafizam Gray-level co-occurrence matrix bone fracture detection American Journal of Applied Sciences 2011 8 26 32 10.3844/ajassp.2011.26.32

[B42] Chai HY Wee LK Swee TT Hussain S GLCM based adaptive crossed reconstructed (ACR) k-mean clustering hand bone segmentation Book GLCM based adaptive crossed reconstructed (ACR) k-mean clustering hand bone segmentation 2011 City: World Scientific and Engineering Academy and Society (WSEAS) 192 197 (Editor ed.^eds.). pp. 192-197

[B43] Gonzalez R Woods R Digital Image Processing 2007 3 Prentice Hall

[B44] Glasbey CA Jones R Fast computation of moving average and related filters in octagonal windows Pattern Recognition Letters 1997 18 555 565 10.1016/S0167-8655(97)00045-7

[B45] Pratt WK Generalized Wiener Filtering Computation Techniques Computers, IEEE Transactions on 1972 C-21 636 641

[B46] Harwood D Subbarao M Hakalahti H Davis LS A new class of edge-preserving smoothing filters Pattern Recognition Letters 1987 6 155 162 10.1016/0167-8655(87)90002-X

[B47] Zhou W Bovik AC A universal image quality index Signal Processing Letters, IEEE 2002 9 81 84 10.1109/97.995823

[B48] Zhou W Bovik AC Sheikh HR Simoncelli EP Image quality assessment: from error visibility to structural similarity Image Processing, IEEE Transactions on 2004 13 600 612 10.1109/TIP.2003.819861 15376593

[B49] Zhou W Bovik AC Mean squared error: Love it or leave it? A new look at Signal Fidelity Measures Signal Processing Magazine, IEEE 2009 26 98 117

